# The Associations between *Helicobacter Pylori* immunoglobulin G seropositivity and body mass index in adults

**DOI:** 10.1186/s12879-023-08427-1

**Published:** 2023-07-20

**Authors:** Jinke Huang, Kunli Zhang, Fengyun Wang, Xudong Tang

**Affiliations:** Institute of Digestive Diseases, Xiyuan Hospital of China Academy of Chinese Medical Sciences, Beijing, China

**Keywords:** *Helicobacter Pylori*, immunoglobulin G, body mass index, associations, adults

## Abstract

**Objectives:**

Inconsistent evidence currently exists regarding the associations between *Helicobacter Pylori* (*H. pylori*) infection and body mass index (BMI). The goal of the current study was to examine independent associations of *H. pylori* immunoglobulin G (IgG) seropositivity and BMI in a U.S.-based population sample.

**Methods:**

The US National Health and Nutrition Examination Survey (NHANES) with 2,576 subjects from 1999 to 2000 were analyzed. Using multivariate logistic regression models, associations between *H. pylori* IgG seropositivity and BMI were calculated after potential confounders were taken into account. Subgroup analyses were conducted furtherly stratified by sex, age, and race.

**Results:**

*H. pylori* IgG seropositivity was not associated with BMI in the general population (OR = 0.998; 95% CI = 0.977–1.019; *P* = 0.842). In the subgroup analyses stratified by race, a negative correction was found between the *H. pylori* IgG seropositivity and BMI among other races (OR = 0.873; 95% CI = 0.795–0.959; *P* = 0.004) except non-Hispanic white (OR = 1.006, 95% CI 0.966 to 1.048, *P* = 0.762), non-Hispanic black (OR = 1.021, 95% CI 0.979 to 1.065, *P* = 0.335), and Mexican American (OR = 1.010, 95% CI 0.966 to 1.055, *P* = 0.665).

**Conclusions:**

In the general population, *H. pylori* IgG seropositivity is not associated with increased BMI, which provides a new perspective on obesity management.

## Introduction

*Helicobacter Pylori* (*H. pylori*) infection is a common, usually lifelong, infection that is widespread worldwide. It is reported that the overall global prevalence of *H. pylori* infection is about 50% [1], and the prevalence in US is about 35.6% [2]. The gastric mucosa-dwelling *H. pylori* strain has the ability to secrete vacuolus toxins, urea enzymes, and cytotoxin, even though the majority of infected people show no symptoms. Chronic gastritis, peptic ulcers, and gastric cancer are all known to be caused by *H. pylori* infection. *H. pylori* infection is believed to have additional gastrointestinal effects in addition to causing the gastrointestinal disease, such as metabolic syndrome, cardiovascular disease, neurological disorder, and ophthalmic disorder [3].

A major health issue worldwide, especially in developed nations, is obesity [4], which is one of the most significant risk factors for metabolic syndrome and a number of diseases [5]. Due to improved medical care and risk factor management, obese people now live longer than they did in the past, but obesity complications like idiopathic thrombocytopenic purpura, iron deficiency anemia, and vitamin B_12_ deficiency place a greater burden on their lives [6]. A significant association between *H. pylori* infection and body mass index (BMI) or obesity has been established in some recent studies [6–8]; however, other studies came to contradictory conclusions [9–11]. Furthermore, conflicting result of multiple pathogenic mechanisms regarding the association of *H. pylori* infection and obesity have been published [10]. *H. pylori* infection has been reported to affect the production of hormones that regulate hunger, which in turn enhances appetite and contributes to an increase in BMI [12]. However, in the case of *H. pylori* infection, leptin, a hormone that can lead to decreased food intake and BMI, is significantly increased [13]. Additionally, *H. pylori* infection might result in dyspeptic symptoms, which might cause a person to consume fewer calories [10]. Thus, the aim of the present study was to explore the association between *H. pylori* IgG seropositivity and BMI among in a large, nationally representative sample of US adults.

## Methods

### Subjects

**Fig. 1:**
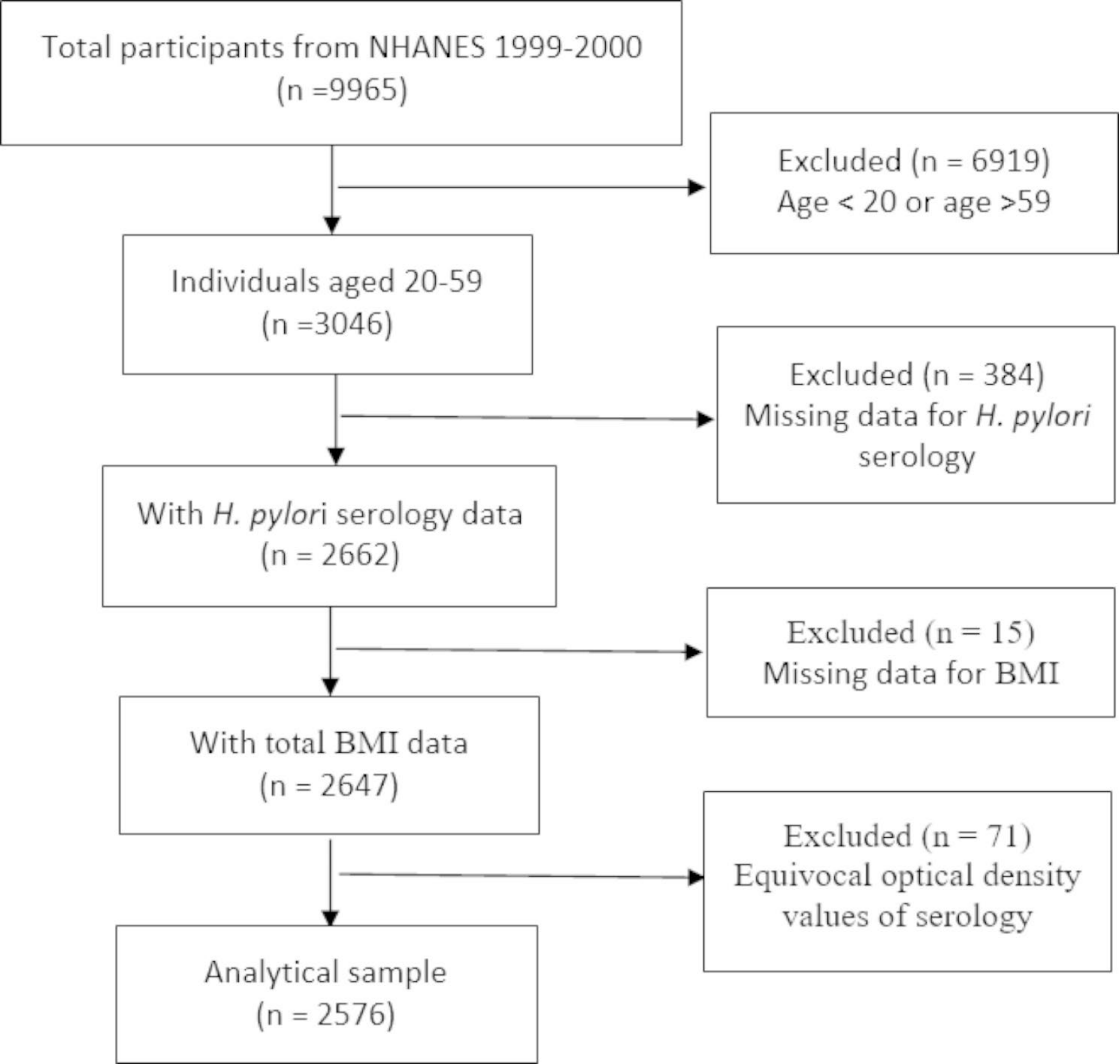
Flow chart of study populations

Data was collected from the 1999–2000 cycle of NHANES in the present study. Since 1960, the US Centers for Disease Control and Prevention have been conducting the NHANES, a nationally representative cross-sectional survey and physical examination of the civilian, non-institutionalized US population [14]. The current study population for this study was limited to adults with complete BMI and *H. pylori* IgG serum antibody data, based on data from subjects who completed a health questionnaire and health screening. Furthermore, only adults aged 20 to 59 were included. 2,576 subjects total were included for the final analysis out of the 9,965 total subjects chosen using the above method. Figure 1 depicts the flow chart for sample selection.

### Variables

The *H. pylori* IgG enzyme-linked immunosorbent assay (ELISA) (Wampole Laboratories), created and standardized at Vanderbilt University, was used to measure *H. pylori* IgG seropositivity. Immune status ratio values of > 1.1 and 0.9 were used to classify each specimen as either seropositive or seronegative, respectively, while values between 0.9 and 1.1 were considered ambiguous [15]. To avoid misleading statistical results, subjects with ambiguous values were excluded from this study. Obesity (> 30 kg/m^2^), overweight (25–30 kg/m^2^), normal (18.5–25 kg/m^2^), and undernutrition (18.5 kg/m^2^) were defined by BMI, which was calculated as weight in kilograms divided by height in meters squared. As covariates, age, poverty to income ratio, days drink in year, serum uric acid, total calcium, blood urea nitrogen, total protein, serum creatinine, total cholesterol, triglycerides, and plasma glucose were applied as continuous variables; sex, race, educational level, physical activity, smoking behavior, and other disease status were applied as categorical variables. On the website of NHANES (www.cdc.gov/nchs/nhanes/), more in-depth information on the variables used in the current study is available to the public.

### Statistical analysis

The R (http://www.r-project.org, The R Foundation) and EmpowerStats (http://www.empowwerstats.com, X&Y Solutions, Inc., Boston, MA) software were used for statistical analysis. To analyze the associations between *H. pylori* IgG seropositivity and BMI, weighted multivariate logistic regression models were used. The weighted logistic regression model was used to determine the differences between groups for continuous variables, and the weighted chi-square test was used for categorical variables. NHANES is a stratified, complex, multistage probability-based survey that oversamples certain groups, and as such, all participants are assigned analytic weights to account for their unequal sampling probability and non-response [16]. In all analyses, sampling design complexity was taken into account by using two-year interview weights for all sample estimations. Statistical significance level was set at p < 0.05.

## Results

### Characteristics of included subjects

In this study, 2576 people met the inclusion criteria, with 1534 being *H. pylori* IgG seronegative and *1042* being *H. pylori* IgG seropositive. People in these two groups differed significantly (*P* < 0.05) in age, race, educational level, income poverty ratio, physical activity, smoking behavior, diabetes status, hypertension status, total calcium, total protein, and plasma glucose. Baseline characteristics of the study subjects are outlined in Table 1.

**Table 1:** Baseline characteristics of the study subjects

	*H. pylori* IgG - (n = 1534)	*H. pylori* IgG + (n = 1042)	*P* value
Age (years)	37.25 ± 10.71	39.86 ± 10.78	< 0.001
Sex (%)			0.11
Male	47.91	51.43	
Female	52.09	48.57	
Race (%)			< 0.001
Non-Hispanic White	79.01	41.47	
Non-Hispanic Black	7.56	19.49	
Mexican American	3.97	15.60	
Other races	9.46	23.44	
Educational level (%)			0.007
Less than high school	13.90	36.54	
High school	24.39	27.16	
College graduate or above	61.71	36.30	
Ratio of family income to poverty	7.71 ± 2.74	6.77 ± 2.67	< 0.001
Body mass index ((kg/m^2^)	27.88 ± 6.56	27.97 ± 6.52	0.76
Waist circumference (cm)	94.34 ± 16.15	94.07 ± 15.38	0.71
Physical activity (MET-based rank) (100%)			< 0.001
0	13.9	29.6	
1	32.5	32.7	
2	20.8	13.9	
3	32.8	23.8	
Smoking behavior (%)			< 0.001
None	54.4	45.5	
Past	21.3	19.9	
Current	24.3	34.6	
Diabetes status (%)			0.03
None	96.2	94.2	
Yes	3.8	5.8	
Hypertension status (%)			0.013
None	82.5	78.2	
Yes	17.5	21.8	
Days drink in year	77.85 ± 96.17	70.23 ± 97.60	0.15
Total calcium (mg/dL)	9.43 ± 0.40	9.40 ± 0.40	0.05
Serum uric acid (mg/dL)	5.19 ± 1.46	5.25 ± 1.49	0.29
Blood urea nitrogen (mg/dL)	13.30 ± 4.30	13.03 ± 3.93	0.13
Total protein (g/dL)	7.51 ± 0.44	7.60 ± 0.44	< 0.001
Serum creatinine (mg/dL)	1.52 ± 0.44	1.55 ± 0.49	0.095
Total cholesterol (mg/dL)	193.62 ± 38.11	192.61 ± 41.05	0.55
Triglycerides (mg/dL)	134.63 ± 101.52	140.34 ± 106.85	0.21
Plasma glucose (mmol/L)	5.39 ± 0.77	5.52 ± 1.60	0.004

Mean ± SD for continuous variables: *P* value was calculated by one-way ANOVA (normal distribution) and Kruskal–Wallis H (skewed distribution) test. % for categorical variables: *P* value was calculated by weighted chi-square test

### Association between H. pylori IgG seropositivity and BMI

#### Model of multiple regression

**Fig. 2:**
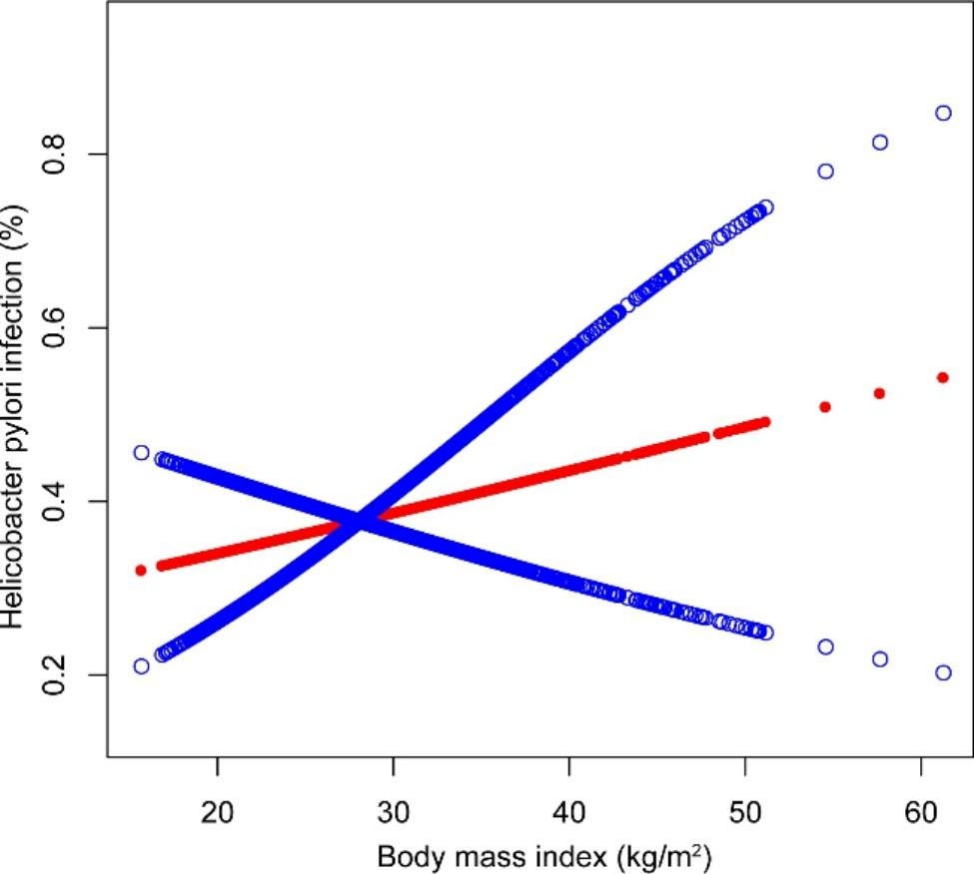
The association between *H. pylori* IgG seropositivity and BMI. Solid red line represents the smooth curve fit between variables. Blue bands represent the 95% of confidence interval from the fit. Age, sex, race, educational level, ratio of family income to poverty, physical activity, smoking behavior, hypertension status, diabetes status, serum uric acid, total calcium, blood urea nitrogen, serum creatinine, total cholesterol, total protein, triglycerides, and plasma glucose were adjusted

*H. pylori* IgG seropositivity was not associated with BMI in any of the multivariable logistic regression models, as shown in Table 2; Fig. 2. After all subjects were divided into four groups according to their BMI, the association between *H. pylori* IgG seropositivity and BMI was not found and there was no significant trend between the different BMI category groups (Table 2).

**Table 2:** Association between *H. pylori* IgG seropositivity and BMI

	Model I, OR (95% CI, *P*)	Model II, OR (95% CI, *P*)	Model III, OR (95% CI, *P*)
Total	1.011 (0.999, 1.023) 0.061	0.995 (0.981, 1.009) 0.461	0.998 (0.977, 1.019) 0.842
BMI categories			
Undernutrition	Reference	Reference	Reference
Normal	1.200 (0.648, 2.223) 0.562	0.749 (0.368, 1.521) 0.424	0.919 (0.329, 2.573) 0.873
Overweight	1.433 (0.774, 2.651) 0.252	0.711 (0.350, 1.447) 0.347	0.902 (0.310, 2.622) 0.849
Obese	1.476 (0.798, 2.732) 0.215	0.676 (0.332, 1.376) 0.281	0.891 (0.280, 2.838) 0.845
*P* for trend	0.023	0.260	0.874

Model I: no covariates were adjusted; Model II: age, sex, and race were adjusted; Model III: age, sex, race, educational level, ratio of family income to poverty, physical activity, smoking behavior, hypertension status, diabetes status, serum uric acid, total calcium, blood urea nitrogen, serum creatinine, total cholesterol, total protein, triglycerides, and plasma glucose were adjusted

#### Subgroup analyses

**Fig. 3:**
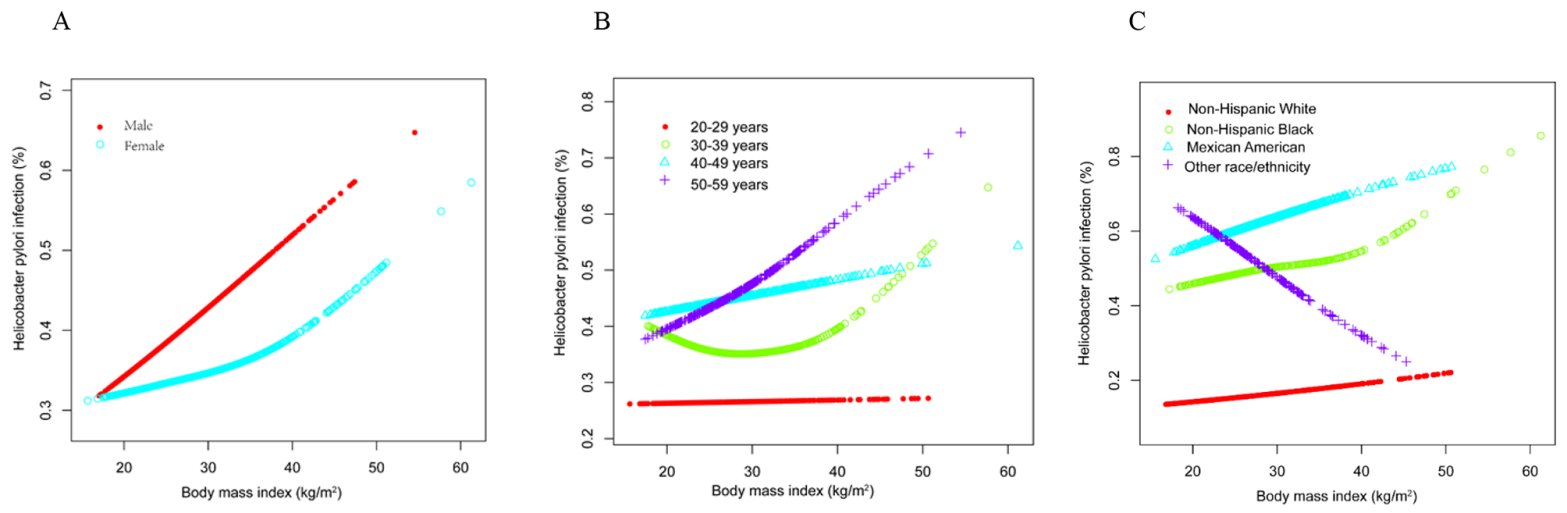
The association between *H. pylori* IgG seropositivity and BMI stratified by sex **(A)**, age **(B)**, and race **(C)**. Educational level, ratio of family income to poverty, physical activity, smoking behavior, hypertension status, diabetes status, serum uric acid, total calcium, blood urea nitrogen, serum creatinine, total cholesterol, total protein, triglycerides, and plasma glucose were adjusted

As shown in Table 3; Fig. 3, when stratified by sex or age, there was no statistically significant association between *H. pylori* IgG seropositivity and BMI. When stratified by race, the association between *H. pylori* IgG seropositivity and BMI was not significant in non-Hispanic white (*OR* = 1.006, 95% CI 0.966 to 1.048, *P* = 0.762), non-Hispanic black (*OR* = 1.021, 95% CI 0.979 to 1.065, *P* = 0.335), and Mexican American (*OR* = 1.010, 95% CI 0.966 to 1.055, *P* = 0.665); however, a negative association between *H. pylori* IgG seropositivity and BMI was observed in other races (*OR* = 0.873, 95% CI 0.795 to 0.959, *P* = 0.004).

**Table 3:** Subgroup analyses of the association between *H. pylori* IgG seropositivity and BMI

	Model I, *OR* (95% CI, *P*)	Model II, *OR* (95% CI, *P*)	Model III, *OR* (95% CI, *P*)
Sex			
Male	1.009 (0.988, 1.030) 0.397	0.995 (0.972, 1.019) 0.692	0.996 (0.961, 1.033) 0.838
Female	1.015 (1.000, 1.030) 0.046	0.995 (0.979, 1.012) 0.570	0.999 (0.972, 1.026) 0.936
Age			
20 ~ 29	0.988 (0.963, 1.015) 0.381	0.982 (0.954, 1.010) 0.200	0.992 (0.945, 1.042) 0.749
~ 39	1.003 (0.980, 1.027) 0.771	0.986 (0.959, 1.013) 0.301	1.001 (0.960, 1.043) 0.970
~ 49	1.020 (0.998, 1.043) 0.080	0.999 (0.974, 1.025) 0.957	0.980 (0.937, 1.024) 0.364
~ 59	1.005 (0.978, 1.032) 0.739	1.011 (0.981, 1.043) 0.479	1.027 (0.970, 1.087) 0.359
Race			
Non-Hispanic White	0.996 (0.971, 1.021) 0.726	0.988 (0.962, 1.014) 0.355	1.006 (0.966, 1.048) 0.762
Non-Hispanic Black	1.004 (0.983, 1.027) 0.691	1.006 (0.983, 1.030) 0.616	1.021 (0.979, 1.065) 0.335
Mexican American	1.014 (0.988, 1.042) 0.295	1.006 (0.979, 1.035) 0.658	1.010 (0.966, 1.055) 0.665
Other races	0.969 (0.932, 1.008) 0.116	0.956 (0.916, 0.998) 0.038	0.873 (0.795, 0.959) 0.004

Model I: no covariates were adjusted; Model II: age, sex, and race were adjusted; Model III: age, sex, race, educational level, ratio of family income to poverty, physical activity, smoking behavior, hypertension status, diabetes status, serum uric acid, total calcium, blood urea nitrogen, serum creatinine, total cholesterol, total protein, triglycerides, and plasma glucose were adjusted

## Discussion

The present study used data from the NHANES to investigate whether there are any independent relationships between *H. pylori* IgG seropositivity and BMI. In summary, *H. pylori* IgG seropositivity was not associated with BMI in the general population. Except for non-Hispanic white, non-Hispanic black, and Mexican Americans, a negative association between *H. pylori* IgG seropositivity and BMI was found in the subgroup analyses stratified by race.

There is conflicting and scant evidence that *H. pylori* infection and BMI are related. A cross-sectional epidemiological study that performed in USA have found no association between *H. pylori* infection and BMI among adults [10], which is similar to the results of our study. Nevertheless, there is a difference, that is, we performed subgroup analysis according to race and found a negative association between *H. pylori* IgG seropositivity and BMI in other races. The findings of a case-control study in the USA, which included 60 participants, revealed that *H. pylori* infection tended to be more common in obese people [17]. Controversial findings have also been found in studies conducted outside of the United States. A positive correlation between *H. pylori* infection and BMI among Chinese people was discovered by a cross-sectional epidemiological study [8] and a meta-analysis [6]. Another study [7] performed in Japan have found similar results. In Taiwan, people were found to have an inverse relationship between *H. pylori* infection and obesity [18], and it has been suggested that *H. pylori* infection eradication may cause a marked increase in BMI [19]. Nevertheless, these studies were performed in East Asian countries. It has been reported that *H. pylori* infection tends to be more common in obese people, and eradication rates are significantly lower in overweight patients than in controls, according to studies [20, 21] conducted in European nations. The eradication of *H. pylori* infection, however, may cause a significant rise in BMI, according to another study [22]. Additionally, according to some studies [9, 11], there is no connection between *H. pylori* infection and BMI, which is essentially in line with the results of our study. Disparities in study design, subject populations, sample size, and *H. pylori* I infection testing procedures may result in inconsistency of the current evidence. Furthermore, because *H. pylori* infection is linked to ethnicity and socioeconomic status, both of which are linked to BMI, studies examining the link between *H. pylori* infection and BMI must accurately and in large enough numbers to allow for adequate adjustment [10]. We clearly discovered there is no correlation between *H. pylori* IgG seropositivity and BMI in this study by analyzing a sizable sample of the representative US population using a rigorous sampling design, high-quality research measurement, and detailed quality control procedures database. Our further subgroup analysis results also showed that a negative association between *H. pylori* IgG seropositivity and BMI was observed in other races except non-Hispanic white, non-Hispanic black, and Mexican American.

For the connection between *H. pylori* infection and BMI, numerous pathogenic mechanisms have been put forth. Obese patients were found to have decreased polymorph nuclear bactericidal capacity [23], decreased ability of monocytes to mature into macrophages [24], and significantly reduced activity of natural killer cells [25]. A more favourable immune environment for *H. pylori* infection may be caused by these immunity changes seen in people with obesity or an elevated BMI [8]. It was also found that *H. pylori* infection can result in insulin resistance and abnormal lipid metabolism by stimulating the release of pro-inflammatory cytokines, ultimately causing obesity or elevated BMI [26, 27]. The production and release of the stomach-derived hormone ghrelin, which increases appetite and contributes to obesity, have also been reported to be affected by *H. pylori* infection [12]. However, in the case of *H. pylori* infection, leptin, a hormone that can lead to decreased food intake and BMI, is significantly increased [13]. Additionally, *H. pylori* infection might result in dyspeptic symptoms, which might cause a person to consume fewer calories [10]. As a result, obesity and *H. pylori* infection may interact, and a large complex may form between them [8]. The prevention and treatment of both *H. pylori* infection and obesity may ultimately benefit from further clarification of the mechanisms underlying the association.

The strength of the study was the use of a large, nationally representative sample of adults. However, some limitations are worth noting. First, the results of this study are based on cross-sectional analysis. Therefore, the temporal relationship between the variables and the causal relationship of the reported associations could not be determined. Second, the choice of confounders or unidentified factors cannot be completely ruled out. Even if we try to reduce the effect of confounders by adjusting for major demographic factors, socioeconomic, and health-related factors. Third, only the subjects of US were included in this study, the conclusions of this study therefore do not apply to patients outside of the US. Finally, a serological test was used to assess *H. pylori* IgG seropositivity. Serological tests have, in general, a limited diagnostic accuracy. Furthermore, we did not test interactions, which is one of the limitations of this study.

## Conclusion

In the general population, *H. pylori* IgG seropositivity is not associated with increased BMI, which provides a new perspective on obesity management.

## Data Availability

The datasets analyzed in this study are available in the NHANES repository (https://wwwn.cdc.gov/nchs/nhanes/Default.aspx).

## References

[1] Zamani M, Ebrahimtabar F, Zamani V (2018). Systematic review with meta-analysis: the worldwide prevalence of Helicobacter pylori infection. Aliment Pharmacol Ther.

[2] Burucoa C, Axon A (2017). Epidemiology of Helicobacter pylori infection. Helicobacter.

[3] Santos Maria Luísa Cordeiro, de Brito Breno Bittencourt, da Silva Filipe Antônio França. Helicobacter pylori infection: beyond gastric manifestations. World J Gastroenterol. 2020;26:4076–93. 10.3748/wjg.v26.i28.4076 PMC7403793 32821071

[4] Zukiewicz-Sobczak W (2014). Obesity and poverty paradox in developed countries. Ann Agric Environ Med.

[5] Grundy SM, Brewer HB, Cleeman JI, Smith SC, Lenfant C (2004). Definition of metabolic syndrome: report of the National Heart, Lung, and Blood Institute/American Heart Association conference on scientific issues related to definition. Circulation.

[6] Xu Xinlan L, Weide Q, Lan (2019). Relationship between Helicobacter pylori infection and obesity in chinese adults: a systematic review with meta-analysis. PLoS ONE.

[7] Hamada Masakazu, Nomura Ryota, Ogaya Yuko. Potential involvement of Helicobacter pylori from oral specimens in overweight body-mass index. Sci Rep. 2019;9:4845. 10.1038/s41598-019-41166-5 PMC6425031 30890723

[8] Xu C, Ming Y, Sun Y et al. Prevalence of Helicobacter pylori infection and its relation with body Mass Index in a Chinese Population. Helicobacter, 2014, 19(6). 10.1111/hel.12153 25256639

[9] Kyriazanos ID, Sfiniadakis I, Gizaris V (2002). The incidence of Helicobacter pylori infection is not increased among obese young individuals in Greece. J Clin Gastroenterol.

[10] Ioannou GN, Weiss NS, Kearney DJ (2005). Is Helicobacter pylori seropositivity related to body mass index in the United States?Aliment. Pharmacol Ther.

[11] Pundak OY, Olivestone CT, Hofi L et al. Lack of association between Helicobacter pylori infection and childhood overweight/obesity. Helicobacter, 2020, 25(5). 10.1111/hel.12728 32686284

[12] Murray CD, Kamm MA, Bloom SR, Emmanuel AV (2003). Ghrelin for the gastroenterologist: history and potential. Gastroenterology.

[13] Breidert M, Miehlke S, Glasow A (1999). Leptin and its receptor in normal human gastric mucosa and in Helicobacter pyloriassociated gastritis. Scand J Gastroenterol.

[14] Dillon CF, Weisman MH (2018). US National Health and Nutrition Examination Survey Arthritis Initiatives, Methodologies and Data. Rheumatic Dis Clin North Am.

[15] CDC. Helicobacter pylori ELISA measurement in NHANES. 2008. Accessed Jan 1st, 2020.

[16] Kantor ED et al. “Trends in Dietary supplement use among US adults from 1999–2012.” JAMA vol. 316,14 (2016): 1464–74. 10.1001/jama.2016.14403 PMC5540241 27727382

[17] Renshaw AA, Rabaza JR, Gonzalez AM, Verdeja JC (2001). Helicobacter pylori infection in patients undergoing gastric bypass surgery for morbid obesity. Obes Surg.

[18] Wu MS, Lee WJ, Wang HH, Huang SP, Lin JT (2005). A case-control study of association of Helicobacter pylori infection with morbid obesity in Taiwan. Arch Intern Med.

[19] Yang YJ, Sheu BS, Chang WL, Cheng HC, Yang HB (2009). Increased body mass index after H. pylori eradication for duodenal ulcer predisposes to erosive reflux esophagitis. J Clin Gastroenterol.

[20] Abdullahi M, Annibale B, Capoccia D, Tari R, Lahner E, Osborn J (2008). The eradication of Helicobacter pylori is affected by body mass index (BMI). Obes Surg.

[21] Arslan E, Atilgan H, Yavasoglu I (2009). The prevalence of Helicobacter pylori in obese subjects. Eur J Intern Med.

[22] Lane JA, Murray LJ, Harvey IM, Donovan JL, Nair P, Harvey RF (2011). Randomised clinical trial: Helicobacter pylori eradication is associated with a significantly increased body mass index in a placebo-controlled study. Aliment Pharmacol Ther.

[23] Palmblad J, Hallberg D, Engstedt L (1980). Polymorphonuclear (PMN) function after small intestinal shunt operation for morbid obesity. Br J Haematol.

[24] Krishnan EC, Trost L, Aarons S, Jewell WR (1982). Study of function and maturation of monocytes in morbidly obese individuals. J Surg Res.

[25] Moulin CM, Marguti I, Peron JP, Rizzo LV, Halpern A (2009). Impact of adiposity on immunological parameters. Arq Bras Endocrinol Metabol.

[26] Basso D, Plebani M, Kusters JG (2010). Pathogenesis of Helicobacter pylori infection. Helicobacter.

[27] Glass CK, Olefsky JM (2012). Inflammation and lipid signaling in the etiology of insulin resistance. Cell Metab.

